# Transactions of the Illinois State Medical Society, for the Year 1855

**Published:** 1856-04

**Authors:** 


### BOOK NOTICES.

*Transactions of the Illinois State Medical Society, for the year* 1855. Peoria Democratic Press Office, Peoria Ill.

Since the issue of the March number of the *Journal*, we have received from the Secretary of the State Medical Society, a copy of the past year's Transactions. We regret exceedingly that the copies are distributed so late in the season; it being now only two months before the next annual meeting. Still it is perhaps better late than never. The published Transactions contain, besides the record of proceedings at the Annual Meeting, in Bloomington, and the reports of the Publishing Committee and Treasurer, the following papers, viz.: "Report of the Committee on Surgery," by J. W. Freer, M.D., of Chicago; "Report of the Committee on Practical Medicine," by E. R. Roe, M.D., of Bloomington; "Report of the Committee on Drugs and Medicines," by H. A. Johnson, M.D., of Chicago; and the Valedictory Address of the President, by C. N. Andrews, M.D., of Rockford; the whole making a pamphlet of 88 pages.

The report of the Committee on Surgery is very brief, occupying less than six pages. But as the Chairman of the Committee, Dr. Freer, was continued in the same position, we presume the present paper will only hold the place of an introduction to a more general and complete report at the next annual meeting. The report of the Committee on Practical Medicine, by Dr. Roe, occupies fifteen pages; a considerable portion of which, consists of interesting extracts from letters received by the Chairman in reply to his circular asking for information. The principal topics discussed in the report are, Typhoid Fever, Typhoid Pneumonia, Scarlet Fever, Erysipelas, and Cholera. In regard to the first named disease, the author of the report collected a sufficient number of facts to show that this form of fever had been quite prevalent during the year 1854, throughout the middle and northern parts of the State; but his attention has been chiefly directed to the influence of mercurials in its treatment. On the latter subject, the reporter says: "Nevertheless, it is still the opinion of the undersigned, that an *early salivation to a slight degree*, will generally cut short the disease." And this he holds as not at all incompatible with the belief, that a bad salivation after the disease is fully developed, will generally aggravate the symptoms. "But it should ever be borne in mind, that by a slight salivation is only meant a *stimulation* of the glands, and not *ulceration*, by any means. And this, to be successful, must be accomplished during what may be called the formative stage of the disease." While such is the opinion of the chairman of the committee, nearly all those practitioners from whom he received communications expressed opinions directly the reverse; declaring, in the language of Dr. S. W. Noble, of Leroy, that they "do not consider the mercurial influence capable of exerting any control over the *course* of the disease." On the subject of Scarlet Fever, the report contains only the following extract from a letter, by Dr. Haller, of Vandalia. We quote it simply for its testimony in relation to the prophylactic powers of the belladonna. "It (the Scarlatina) began to prevail," says Dr. Haller, "in the latter part of Autumn, and forepart of Winter. In the beginning it was very malignant in character, some few sinking in a few hours; and many of those who recovered were well nigh exhausted from suppuration, ascites, &c. About the middle of the epidemic, it became much milder; after which, not a single case proved fatal, which I attributed to the prophylactic effects of the belladonna which I had been giving to all that I attended subsequently. In relation to the prophylactic powers of the belladonna, adds Dr. Haller, I think it will not prevent persons from having Scarlatina; but, of one thing I am satisfied—that it will modify it *equal to the modification of Small Pox by vaccination.*" On the subjects of Cholera and Erysipelas, the report is also very brief. During the year 1854, the former disease prevailed extensively in most of the towns and cities in this State. It was particularly severe in this city; and a pretty full history of its character and progress was published in the pages of the *Journal:* but the Committee make no allusion to the matter in their report.

With no disposition to be either critical or censorious, we cannot refrain from expressing the opinion, that the report, in all its departments, is exceedingly defective.

The report of the Committee on Drugs and Medicines occupies twenty-three pages, thirteen of which are made up of letters from practitioners in different parts of the State, written in answer to a circular sent out by the chairman of the Committee.

Both the report of the Committee, and the letters appended thereto, contain matter of interest to the Society and the profession; and yet we find but little of practical value which has not already appeared, from time to time, in the pages of the *Journal.*

The last paper in the Transactions, is the President's Address.

The theme is, "Medical Progress;" and had we space we would copy it entire. As it is, however, our readers must be content with the following extracts; unless they do what would be far better, viz.: procure of the Secretary of the State Society a copy of the Transactions and read the whole.

"The subject of progress, in general," says Dr. Andrews, "is one upon which we hear much said at the present day. The world, at least the American part of it, seems to be in a partial frenzy upon it. We hear the word progress on almost every tongue. Its echoes reach us from every department of moral, social, and industrial life. It is the child's first lesson; it is the youth's first task. Ripe manhood makes an occupation of it; and mature years rejoice over its practical and beneficial results, while the octogenarian 'pipes and whistles' over its novelties and wonders.

"That this is truly an age of progress, it is not necessary to attempt to prove, for we see enduring monuments and forcible illustrations of it on every hand. But in the very foreground of all these things, we have the most indubitable evidence that all is not progress, at the present day, that assumes to be such. Indeed, some of the rankest and most mischievous errors that ever infected society, or the human mind, are flourishing under its popular garb. And, furthermore, the prevailing idea that this is a golden age of progress, proves fatal to many individuals; they descant largely upon it, feed and grow fat upon it, and never take the pains to learn even first principles, and ultimately die stupidly ignorant. And this is the case with many physicians. They rejoice in the great achievements of medicine, and glory over its brilliant truths; and they live upon those ideas, and are lulled by them into a luxurious indolence, and a pleasing anticipation of a period when our achievements will be so great that quacks will not be able to dwell in the land."

Again he says:—

"True *progress* consists in adding that which is new and useful of the present, which has been ascertained, developed, or achieved by observation, experience or philosophical or experimental research, to that which is useful of the past, and, as occasion may require, in substituting that which is new of the present, for so much of the old as reason and experience may teach to be advantageous. The substitution being made not because the old is inherently or necessarily bad or vicious, but because the new possesses some qualities of value which the old does not."

The author defines clearly the difference between progress and reform. The latter, he claims, is related exclusively to *moral* changes; to something which affects "the heart, the conscience." Consequently, the term can have no application to matters of science and literature. Hence, he says, "That which is vicious or morally corrupt, may be *reformed;* but a science or an art, however imperfect it may be, can only be improved upon by *progress;* which latter must be the fruits of experience and discovery." The mental traits essential for making progress are stated as follows:—

"Progress in medicine depends upon these qualities or conditions, viz.: mental capacity or qualification; a consciousness of a necessity for it, and the will, resolution, and energy, to so conduct the details of our transactions that they may all be conducive to this end; and, lastly, unity of effort among the members of the medical profession."

Dr. Andrews comments with considerable severity on the prevailing tendency to discard the study of ancient authors, and the insatiable longing after the latest and most popular, even in medicine. He claims for the ancient medical writers, the same position in relation to the student of medicine, as is held by Xenophon and Plutarch, Horace, Cicero, and Virgil, in the education of the student of general science and literature. In illustrating the importance of this, he dwells somewhat in detail on the mental characteristics of Hippocrates, Galen, Bœrhaave, Cullen, and Rush, together with their modes of studying and improving medical science. The following lengthy extract constitutes the closing pages of the address, and will be read with interest:—

"But we have now arrived at the period in the course of medical time, when we begin to see the medical mind developed in our own country, and duty to ourselves and to our science, as well as the sentiment of patriotism, forbid that it should be passed over in silence, although it would be in exact accordance with the custom of the day. For it has become so unfashionable and seemingly unlearned, to refer to the early physicians of this country, unless by the way of derision, that few have the temerity to do it.

"The early physicians of America were generally men of remarkable character, particularly those who lived during the latter part of the eighteenth century. They, as a general rule, had been well educated, and the peculiar scenes and circumstances with which they were surrounded, seemed to give them great energy and independence of mind. For awhile they bowed to foreign authority, as obsequiously perhaps as some of their descendants now do; but this, in those days, was to be expected, for, as a matter of course, they had been educated in the old country, and there had obtained not only their science, but their opinions also. However, not many generations had passed away when the medical mind had become as free as the political mind, and as competent to perform great deeds. At the time of the war of the revolution, we had physicians who, in ability, have never been surpassed. They possessed an intelligence, a solidity of thought and a philosophical strength that have rarely been equalled, and their success in practice, I am confident, has never been excelled. Their writings, in proportion to their extent, possess a value which is without comparison, and they should be deligently read by every American physician. But, sad to relate, they are rapidly passing from the memory of man, almost out of print, and must soon sink into comparative oblivion, to our eternal shame, unless we cease to present our professional offerings to a foreign Moloch, and exhume our own peculiar medical literature, and cultivate it as such.

"Is it generally known to the mass of American physicians, Dr. BENJAMIN RUSH wrote thirty-six distinct treatises on different medical subjects? And how many are there who read them, and appreciate their value; who ever give his important doctrines a serious or attentive thought; who ever look to him for a principle or a precept; or who ever attempt to obtain a lesson from his eventful life? Again, let me ask how many, at the present day, profit by the almost incomparable experience and teachings of Dr. NATHAN SMITH? And I might extend the same inquiries to the writings of Mitchell, the Millers, Caldwell, Ramsey, Warren, Physic, Bigelow, and a number of others.

"But the writings of these fathers of American medicine, are in a manner 'buried and lost,' and with them the only true basis of American literature. We must do our thinking and investigating, and we must be guided by our own philosophy. So long as we are satisfied with merely imbibing the froth and scum of medical science, which floats to us through the medical press from the other side of the Atlantic, so long will our native energies remain, to a great extent, inactive; so long will we remain without a literature characteristic of ourselves, or which will represent in a favorable light the medical mind and talent of the nation; and so long shall we remain comparatively destitute of ample medical libraries. Without literature, and without libraries, judicious physicians will never feel themselves competent to write for the medical public, and their observations and experience, however extensive and valuable, must die with their physical bodies; and editors of medical journals, when they have called in vain for communications, will have to fill out their columns, as usual, with extracts from foreign matter, and necessarily diluted editorials.

"It has been justly remarked, by Frederick Schlegel, a distinguished German writer, that 'every separate and independent nation has the right to possess a literature peculiar to itself.' He of course referred to general literature; but does not the remark apply as well to medical literature? How can medical science be cultivated by a nation unless it has a literature of its own? Without this, its medical mind must be feeble and diminutive, or the mere reflection of that of some other country. It is a lamentable truth that we do not possess a medical literature that bears the impress of originality and independence—the innate sagacity and stubborn philosophy of the American character exhibited in other departments of science and literature. Is there not some obvious cause for this state of things? A modern writer of medical history remarks, that 'for centuries all the improvements in medicine which were even contemplated, consisted of but little more than illustrations of the doctrine of Galen, or commentaries on his writings.' And whoever continues the history of medicine will have to write the history of a similar, though I hope, shorter period, by adding, at least, that for a period of nearly fifty years, ending about the middle of the nineteenth century, the physicians of America did but little more than 'illustrate' and 'comment' upon the medical dogmas received from Europe. Again, our medical students, as a general rule, do not receive an adequate preliminary education. Indeed, there is not as much attention paid to this as there was a half century ago; they are taught medicine more as an art than as a science, founded on a broad and far reaching basis. Our teachers, with some very honorable exceptions, are not men of deep learning or research. Our medical journals that are mostly patronized, are mere reprints of foreign publications. Our authors, with few exceptions, are, in the language of Sprengel, 'mere frigid compilers;' they are mere chroniclers of other men's ideas, and hirelings of book publishers. They import their opinions from a foreign market, and clothe themselves in the cast-off garments of foreign authors, and verily may be characterized as *sui generis*, a cross between the ape and the snob. And it has come to be the case, that if an American physician attempts to write an original work, without adopting a foreign dress, model, or system, his own brethren either 'damn him with faint praise,' or criticise him to death. I would like to run through the list of our medical works, and show what servility is paid to foreign writers, but time would not permit. However, I cannot do less than name an instance or two, meaning no disrespect to the authors, and only selecting these particular works because they have attracted most attention, and are, perhaps, among the most learned and ingenious specimens of our literature. The work of Dr. Meredith Clymer, on fever, is made up almost entirely from the contributions of Drs. Christison, Shapter, Burrows, Gregory, and Locock, to 'Tweedie's Library of Practical Medicine,' when abundant materials, in my humble opinion, far better, might have been found at home. And the writings of Drs. Bartlett and Flint, on fever, are but little more than 'illustrations and commentaries' on the dogmatic pathology of Breteneau and Louis. Now, it seems to me, that these authors, and others of a kin, have sold their birthright for a mess of pottage; have left a rich inheritance of truth, to quaff the merest foibles of foreign theorists, and have left their own native medical literature, and sound knowledge, to sport in the freaks and fashions of foreign capitalists.

"For such reasons, the medical mind in this country seems, in some respects, to have deteriorated, to have lost much of its independent action and pristine energy. There was a time in our history when our physicians did not so servilely follow everything that was foreign. When the yellow fever broke out in this country, about the commencement of the present century, several foreign physicians commenced writing upon it; several in France, one in Ireland, and, finally, Dr. Haygarth, of England. The latter wrote a book upon it, and sent a copy of it to the College of Physicians at Philadelphia, for the purpose of instructing them in the prevention and extermination of the American pestilence, as it was then called. A letter, in reply, was immediately thereafter sent to England, giving an expression of the sentiments of American physicians, touching the gratuitous services of Dr. Haygarth and his co-laborers. This letter is worthy of immortality, and it should be attentively studied by all our medical snobs, both of this and of all future generations. I can only quote a single passage from it. The writer says: 'Although the American States can no longer be viewed as colonies or provinces dependent upon the politics or government of sovereign Europe; still the schools of that quarter of the globe claim America as a district of their literary empire, and usurp scientific dominion over the minds of her inhabitants. They pretend, that they must see for them, and hear for them, and feel for them, and not they for themselves. They affect to gather facts for them, to make experiments for them, to reason for them, and to judge for them, lest through their extreme imbecility they should go astray.' 'But,' the writer further remarks, 'it is time for Americans to abjure publicly all such supremacy, and to assert their own dignity and privilege; to declare that on the ground of free, fair, and equal discussion, they will meet them as philosophical friends; but to proclaim that they will exercise their senses, their understandings, their judgments, free from all authority, dogmatism, or any other control, save truth alone.' He further remarks: 'Is there some hebetude of intellect, some unhappy defect of capacity, which distinguishes and degrades the man of the West? We shall not affirm positively that this is not the case, though we certainly have no evidence to convince us that it is. On that point we shall be content to let their comparative merits be judged of by others.' The only difference in the two instances of literary despotism, is, that, at the present time, it is self-imposed.

"It is very true, that we have some fearless and independent talent at the present day; and I think, of this, the West has her full share. But its struggles against the tides are truly hard and perplexing. To give some idea of this, I will present a few instances near home. Prof. BRAINARD, of Chicago, had the resolution to presume to give the Academy of Sciences, of Paris, some months since, a lesson on the treatment of poisoned wounds. It seems to have been well received, and created some sensation over the water. But on this side it could scarcely be credited. It seemed so improbable, that it was thought that there must be some mistake in the name. So Dr. BRAINARD'S name was mutilated and rendered peculiarly French, and he transformed into a Frenchman, residing at Paris; and thus he became '*M. Benard*,' and was made to go the rounds of our newspapers as the author of the paper above referred to. Again, Dr. BENNETT DOWLER, of New Orleans, had the audacious independence to dissect a live alligator, for the purpose of testing the accuracy of some modern physiological doctrines; and, forthwith, a representative of the English school of physiologists came to America to look to their laurels; but what is to be particularly regretted in the case, is, that Dr. DOWLER'S efforts have been passed over by most of the medical press of this country, as unprofitable and useless innovations, and not worthy of serious consideration. Another case in point, which I wish particularly to notice, because it more immediately concerns us in our general practice, is this:—Not long since, there appeared in the *North-Western Medical and Surgical Journal*, several articles on the subject of fever, written by Prof. DAVIS, of Chicago. In those articles, the author advanced some very important ideas bearing upon what I wish to call the *American doctrine of fever*, the *unity* of fever as it is termed; which doctrine, allow me to say, I regard as truthful as nature itself. It has been advanced and supported by the most able and distinguished of American physicians, and first of all by Dr. BENJAMIN RUSH. It is supported by facts which are incontrovertible, and in these articles of Prof. DAVIS', a fund of new argument is brought to their support. Yet the followers of the 'little gland,' and nosological schools can see no force in his arguments, and they regard the doctrine as a homespun utopianism, and not worthy of their attentive consideration. And let me add, that this is the fate of nearly every thing written by Americans of original and independent thought.

"But why does such a state of things exist? It is because the medical mind of this country has become so completely entangled with foreign doctrines, especially with regard to practical medicine, that it has become in a manner paralyzed—its vision distorted, and that it acts merely from habit or by imitation.

"But let it not be understood that we are blind to the great value of foreign medical literature, or that we underrate the improvements and discoveries that are continually being made in the medical sciences in England, and on the continent of Europe, for we are all aware that we could not well do without them. But because we have received a rich legacy from this source, and expect to receive more, we should not act the part of the rich man's son, who having been brought up in wealth and luxury has contracted the vices incident thereto, and stupidly spends his life in the expectation of some day being made great by his patrimony, and finally dies with *dementia* from the gout.

"It may be thought that, in the course of my remarks, I have attached too much importance to the study of medicine, as it has existed in different ages past, that I have counselled a blind adherence to old authority; but let me be clearly understood on this subject. I only advise the study of the past, that we may more clearly understand and appreciate the present, and be better prepared to create a future. As the corner stone of modern philosophy is laid of ancient materials, so modern literature has its foundation in the past; so must medicine rest upon those truths and those qualities of mind which it has required centuries to unfold. We want the wisdom and experience of physicians of different ages of the world, as the mariner wants the chart of those who have sailed before him. We want to have pointed out to us the rocks on which others have split. We want the mind and philosophy of the past, from which we may select materials to enlarge and strengthen our own. And as Schlegel remarks, in regard to Plato and Aristotle, that 'they have so distinctly marked out the two great paths of human thought and science, that they have remained, and always must remain, the master guides of all succeeding generations;' so may we remark in regard to our great fathers in medicine, that they, in like manner, have so distinctly marked out the great paths of medical thought and medical science, that they likewise must be the master guides of all succeeding generations.

"That the ancients were perfect in knowledge and science, we do not pretend; and if there were defects in ancient medicine, so there were in ancient philosophy, but modern science at once detects these and profits even by the detection. That there were errors and imperfections in the Aphorisms of Hippocrates, the Physiology and Commentaries of Galen, the Materia Medica of Aretæus and Dioscorides, the Pathology, Therapeutics, and Surgery of Celsus, the Aphorisms and Institutions of Boerhaave, the Commentaries of Van Swieten, the Ratio Medendi of De Haen, the Methodus Curandi Febres of Sydenham, the Medical and Surgical Memoirs of Dr. Nathan Smith, and the Medical Enquiries and Observations of Dr. Benjamin Rush, is very evident. But still there is a halo of wisdom and inspiration gleaming from these old volumes, from the potent influences of which no physician should be deprived. No student of medicine can sit down and peruse these old dusty pages without having his mind enlarged and moulded to truer form, his zeal strengthened, and without being made a wiser man, a better physician, and withal incalculably better prepared for medical progress. In these old volumes we find every variety of doctrine, it is true, but the promulgators of these have each done their particular work to advance medical science. The Humoral Pathologists did their work in investigating the morbid changes in the body, and in directing the attention of physicians, for all time, to the subject. The Solidists did a like work in investigating the morbid changes in the solids, and in attaching an importance to the subject that will not be forgotten. The Mechanical Pathologists have led us to observe that the animal functions are not wholly beyond the influence of mechanical and physical laws. The Chemical Pathologists have led us to the knowledge that there are chemical affinities which enter into and harmonize with vital action in health, and which prey upon the organs and functions of life, in disease. And those who saw in the human system an *anima*, an *archeus*, or a *vis medicatrix natura*, have directed us onward to most successful research into the laws of vital organization.

"Finally, gentlemen, let us be true to ourselves and to our medical literature, and then we shall be true to our science. Let us be true to ourselves, by taking facts from whatever source they may come, and forming our own opinions. Let us be true to ourselves by rendering available that which is classic in medicine and which is the common inheritance of all men who study the healing art. Let us be true to ourselves by encouraging and sustaining our own medical literature and cultivating it as such. And let us be true to our own fathers in medicine, by giving to their writings that consideration to which they are entitled, and by paying a just tribute to their memory. Great Britain has had her Sydenham, Harvey, and Hunter; France, her Fernel, Lieutaud, and Duret; Germany, her Bœrhaave, Haller and Van Sweiten; Spain, her Lully and Arnoldus; Italy, her Mondini, Baglivi, and Burserius; and Arabia, her Rhazes and Avicenna; and these individuals are sacredly embalmed in the memory of their countrymen, and they are to them living lights in the pathway of medical science. America has had her Rush, her Nathan Smith, and their worthy coadjutors, and shall we be less mindful of their virtues, or pay less regard to their precepts?"

Without endorsing all the sentiments contained in the address of Dr. Andrews, we would earnestly commend its perusal to our readers, with the assurance that they will find in it much that is interesting, and many thoughts which are worthy of careful consideration. We wish we could here end our notice of the Transactions before us, but our duty to ourselves and the Society require a decided protest against the style in which they have been printed. If the proof sheets were read at all, they were never corrected, for the typographical errors are as numerous as they are glaring and inexcusable. A typographical error will now and then occur, in all transient publications, and are scarcely worthy of serious notice; but when they occur at the rate of from one to half a dozen on a page, it betrays too great a degree of carelessness on the part of those whose business it is to read and correct the proof sheets.

The following are the present Officers of the Society, and the Committees from whom reports may be expected at the next meeting, to be held in Vandalia, on the first Tuesday in June.

DR. N. S. DAVIS, Chicago, *President.*

DR. E. R. ROE, Bloomington, F. A. McNEIL, Mt. Morris, *Vice-Presidents.*

DR. J. V. Z. BLANEY, Chicago, *Treasurer.*

DR. E. ANDREW, Peoria, *Secretary.*

DR. PRINCE, Jacksonville, *Assistant Secretary.*

Drs. A. D. STEARNS, —— DODGE, F. B. HALLER, Vandalia, *Committee of Arrangements.*

Drs. SAMUEL THOMPSON, Albion, J. W. SPAULDING, Galesburg, J. O. HARRIS, Ottawa, *Practical Medicine.*

Drs. J. W. FREER, Chicago, D. PRINCE, Jacksonville, A. H. LUCE, Bloomington, *Surgery.*

Drs. F. A. McNEIL, Mt. Morris, F. K. BAILEY, Joliet, J. MORSE, Galesburg, *Obstetrics.*

Drs. H. A. JOHNSON, Chicago, J. BLOUNT, Rockford, T. W. THOMPSON, Albion, *Drugs and Medicines.*

SPECIAL COMMITTEES.

Prof. BLANEY, Chicago, *Toxicology of the Alkaloids.*

DAVID PRINCE, Jacksonville, *Orthopœdic Surgery.*

Dr. F. K. BAILEY, Joliet, *Congestive Intermittents.*

Dr. E. ROE, Bloomington, *Influence of civilization and mental culture as a cause of difficult labor.*

Dr. W. B. HERRICK, Chicago, *Physiological and pathological properties and influence of fibrine.*

Dr. C. R. PARK, *on Pseudarthrosis.*

Dr. A. S. McARTHUR, Joliet, *on the physiological explanation of counter irritation.*

Dr. N. S. DAVIS, Chicago, *on the alterations of blood in continued fever.*

